# Unlocking the Power of Onion Peel Extracts: Antimicrobial and Anti-Inflammatory Effects Improve Wound Healing through Repressing Notch-1/NLRP3/Caspase-1 Signaling

**DOI:** 10.3390/ph16101379

**Published:** 2023-09-28

**Authors:** Rafik Mounir, Walaa A. Alshareef, Eman A. El Gebaly, Alaadin E. El-Haddad, Abdallah M. Said Ahmed, Osama G. Mohamed, Eman T. Enan, Shaimaa Mosallam, Ashootosh Tripathi, Heba Mohammed Refat M. Selim, Sarah I. Bukhari, Rihaf Alfaraj, Ghada M. Ragab, Amira A. El-Gazar, Soad Z. El-Emam

**Affiliations:** 1 Pharmacognosy Department, Faculty of Pharmacy, Misr University for Science and Technology, Giza 12585, Egypt; rafik.farajallah@must.edu.eg; 2 Microbiology and Immunology Department, Faculty of Pharmacy, October 6 University, Giza 12585, Egypt; dr.walaa@o6u.edu.eg (W.A.A.); eman.elgabali.pha@o6u.edu.eg (E.A.E.G.); 3 Pharmacognosy Department, Faculty of Pharmacy, October 6 University, Giza 12585, Egypt; alaa_elhaddad.ph@o6u.edu.eg; 4 Pharmacology and Toxicology Department, Faculty of Pharmacy, October 6 University, Giza 12585, Egypt; abdallahmohammed@o6u.edu.eg (A.M.S.A.); amira.ahmed@o6u.edu.eg (A.A.E.-G.); 5 Pharmacognosy Department, Faculty of Pharmacy, Cairo University, Kasr el Aini St., Cairo 11562, Egypt; osama.mohamed@pharma.cu.edu.eg; 6 Natural Products Discovery Core, Life Sciences Institute, University of Michigan, Ann Arbor, MI 48109, USA; ashtri@umich.edu; 7 Department of Pathology, Faculty of Medicine, Mansoura University, Mansoura 35516, Egypt; emanenan@mans.edu.eg; 8 Department of Basic Medical Sciences, College of medicine, AlMaarefa University, Riyadh 13713, Saudi Arabia; 9 Department of Pharmaceutics and Industrial Pharmacy, Faculty of Pharmacy, October 6 University, Giza 12585, Egypt; 10 Department of Medicinal Chemistry, College of Pharmacy, University of Michigan, Ann Arbor, MI 48109, USA; 11 Department of Pharmaceutical Sciences, Faculty of Pharmacy, Al-Maarefa University, Diriyah, Riyadh 13713, Saudi Arabia; hmustafa@mcst.edu.sa; 12 Microbiology and Immunology Department, Faculty of Pharmacy (Girls); Al-Azhar University, Cairo 11651, Egypt; 13 Department of Pharmaceutics, College of Pharmacy, King Saud University, Riyadh 11451, Saudi Arabia; sbukhari@ksu.edu.sa (S.I.B.); ralfaraj@ksu.edu.sa (R.A.); 14 Pharmacology and Toxicology Department, Faculty of Pharmacy, Misr University for Science and Technology, Giza 12585, Egypt; ghada.ragab@must.edu.eg

**Keywords:** dietary flavonoids, anthocyanins, quercetin, tissue regeneration, angiogenesis, VEGF, *Candida*, MRSA

## Abstract

Onion peels are often discarded, representing an unlimited amount of food by-products; however, they are a valuable source of bioactive phenolics. Thus, we utilized UPLC-MS/MS to analyze the metabolomic profiles of red (RO) and yellow (YO) onion peel extracts. The cytotoxic (SRB assay), anti-inflammatory (Griess assay), and antimicrobial (sensitivity test, MIC, antibiofilm, and SP-SDS tests) properties were assessed in vitro. Additionally, histological analysis, immunohistochemistry, and ELISA tests were conducted to investigate the healing potential in excisional skin wound injury and *Candida albicans* infection in vivo. RO extract demonstrated antibacterial activity, limited skin infection with *C. albicans*, and improved the skin’s appearance due to the abundance of quercetin and anthocyanin derivatives. Both extracts reduced lipopolysaccharide-induced nitric oxide release in vitro and showed a negligible cytotoxic effect on MCF-7 and HT29 cells. When extracts were tested in vivo for their ability to promote tissue regeneration, it was found that YO peel extract had the greatest impact. Further biochemical analysis revealed that YO extract suppressed NLRP3/caspase-1 signaling and decreased inflammatory cytokines. Furthermore, YO extract decreased Notch-1 levels and boosted VEGF-mediated angiogenesis. Our findings imply that onion peel extract can effectively treat wounds by reducing microbial infection, reducing inflammation, and promoting tissue regeneration.

## 1. Introduction

The daily consumption of vegetables and fruits in the Mediterranean diet is usually associated with health benefits. The *Allium* genus, including onions, garlic, and chives, is a main component of the Mediterranean diet whose protective role towards several disorders has been evidenced [1,2]. Onion is the second-most important horticultural crop, accounting for 47 million tons every year [3]. Several studies have documented the efficacy of onion extract as a potent anti-inflammatory and antioxidant agent [4,5]. Nevertheless, there is a scarcity of research pertaining to the efficacy of onion peels. Different colors of onion peels are strictly dependent on flavonoid content; the yellow color is mostly due to quercetin derivatives, while the red color is due to anthocyanins [6]. The disposal of agri-food wastes leads to the unfortunate loss of the valuable nutritional and therapeutic capabilities inherent in their bioactive substances. Therefore, valorizing onion peels by turning them into food by-products that can be used as functional ingredients is required [7]. 

Skin wounds impose a considerable burden on both the economy and society; therefore, they are an important issue in healthcare systems worldwide [8]. The healing process in skin wounds is an intricate physiological event with four sequential phases—hemostasis, inflammation, proliferation, and maturation—to restore normal skin integrity [9]. If the healing process is interrupted, wounds turn chronic and become more susceptible to microbial infections that can hamper and further slow down the healing process, like those observed in diabetes [10]. Wounds have a susceptibility to bacterial infections, such as *Staphylococcus aureus*, as well as fungal infections, such as *Candida albicans*. The toxins generated can potentially impede the process of wound healing through the augmentation of the inflammatory cascade [11,12]. Various studies have elucidated the antibacterial characteristics of red onions, indicating their potential application in addressing microbial infections, namely those that exhibit resistance and impede the timely progression of wound healing [13]. On a molecular basis, following skin damage, the activation of Neurogenic Locus Notch Homolog Protein-1 (Notch-1), a crucial transcription factor involved in the creation and maintenance of the skin, along with the Pyrin Domain Containing Protein 3 (NLRP3) inflammasome, results in the induction of tumor necrosis factor-α (TNF-α) and other inflammatory cytokines [14,15]. Furthermore, the activation of the Notch-1 pathway in fibroblasts leads to a reduction in wound angiogenesis, thereby causing a delay in the healing process [16]. Hence, the strategic focus on the Notch-1/NLRP3/caspase-1 pathway holds significant potential for enhancing wound healing and promoting tissue regeneration.

Contemporary wound healing therapies frequently exhibit inadequate efficacy and are accompanied by a multitude of significant adverse effects. Furthermore, these therapies demonstrate limited effectiveness in combating resistant infections, hence hindering the wound-healing process [17]. The traditional use of plants in wound healing and halting microbial infection offers a widely available, economical, and reliable natural source of therapeutic compounds [18,19]. There is a limited amount of existing studies on the biological activity of onion by-products and their metabolite profiles. A recent study provided documentation on the efficacy of onion peel extract as an antibacterial agent in facilitating the wound healing process [20]. Nevertheless, despite these discoveries, the exact mechanism by which this enhancement takes place is still not well understood. Hence, the aim of the present study was to execute chemical profiling, antimicrobial assessment, and an investigation of the biological activities of onion peels. Additionally, we sought to elucidate the mechanism behind the wound-healing properties of onion peels by examining their influence on the Notch-1/NLRP3/caspase-1 pathway.

## 2. Results and Discussion

### 2.1. Chemical Profiling of Onion Peel Extracts Using UPLC-QTOF-MS/MS

**Figure 1:**
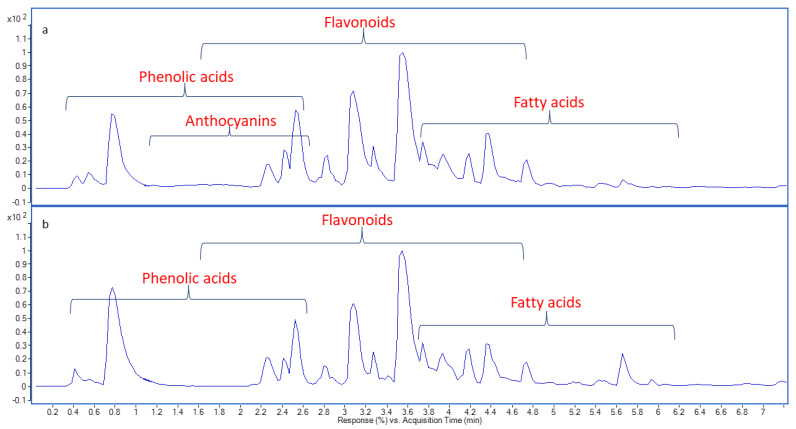
Base peak chromatograms of onion peels; RO (**a**) and YO (**b**) methanol extracts in negative ionization mode.

Polyphenols, flavonoids, and anthocyanins have attracted extensive attention for their beneficial effects on health, such as antioxidant and antimicrobial activities as well as protection against coronary heart disease [21]. The base peak chromatograms of onion peel extracts in negative ionization mode are presented in Figure 1. Metabolite assignments were made by comparing retention time and HRMS/MS data, whenever available, or interpreting MS data combined with the chemo-taxonomic data reported in the literature. As recently reported, quercetin glycosides are predominant in onions, whereas anthocyanins are mainly detected in red onions, where they account for about 10% of the total flavonoid content [22]. Flavonoid; aglycones and *O*-glycosides, anthocyanins, phenolics, and fatty acids were identified. Metabolites (51 and 48) were detected from RO and YO peel extracts, respectively, and showed a comparable qualitative profile, except for the presence of anthocyanins (54–58) in RO peel extract. In both RO and YO peel extracts, flavonols were the most detected compounds, in particular quercetin, kaempferol, isorhamnetin, taxifolin, and myricetin derivatives. No difference in flavonol profiles was observed between the two onion varieties (Figure 1). Both RO and YO peel extracts contained flavonol glycosides, with quercetin di-*O*-hexoside derivatives (28, 30, and 31) and quercetin mono-*O*-hexoside derivatives (37, 41, 44, and 45), in addition to quercetin dimers (47 and 51–53) (Table 1). Quercetin is the most abundant flavonol in the onion peel, according to previous studies [6,22].

**Figure 2:**
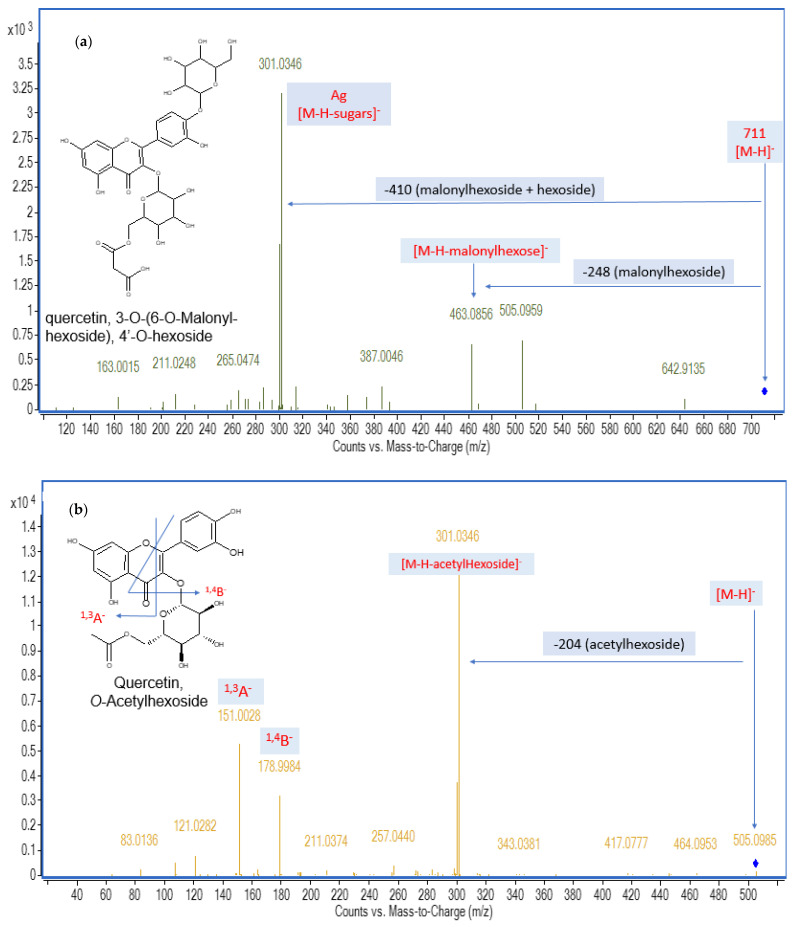
Mass fragments of some identified phytoconstituents in the onion peel extract: (**a**) quercetin, *O*-(*O*-malonyl-hexoside), *O*-hexoside; (**b**) quercetin-*O*-acetylhexoside.

Acetylated quercetin glycosides: A deprotonated molecule at *m*/*z* 667.15 [M−H]^−^ and the neutral loss of 204 amu of acetylhexosyl moiety yielding the product ion at *m*/*z* 463 [M−H−hexosyl acetate]^−^, which showed a subsequent neutral loss of 162 amu of hexosyl moiety, was tentatively identified as quercetin-*O*-hexoside-*O*-acetylhexoside (30). A deprotonated molecule at *m*/*z* 711.1403 [M−H]^−^ and the neutral loss of 248 amu of malonylhexosyl moiety that was observed in MS^2^ fragmentation at *m*/*z* 463 [M−H−malonylhexosyl]^−^ with another neutral loss of 162 amu of hexosyl moiety was proposed as quercetin, *O*-(*O*-malonyl-hexoside), *O*-hexoside (31) (Figure 2a). In the same manner, quercetin-*O*-acetylhexoside (41) (Figure 2b) and *O*-galloylquercitrin (45) were identified.

Quercetin glycosides and dimers: The MS^2^ spectrum of a deprotonated molecule [M−H]^−^ with *m*/*z* 625.1421 showed two subsequent neutral losses of 162 amu, representing the hexosyl moiety yielding the product ions at *m*/*z* 463 and 301, respectively, with the typical fragmentation pattern of quercetin, which was proposed as quercetin-*O*-dihexoside (28). In the same manner, quercetin-*O*-hexoside (37) was detected at *m*/*z* 463.0896 [M−H]^−^. *Allium* quercetin dimers (51–53) were detected by deprotonated molecules at *m*/*z* 601.0625 and the neutral loss of 302 amu of quercetin moiety in MS^2^ fragmentation at *m*/*z* 299; furthermore, the characteristic MS^2^ fragments of quercetin were detected at *m*/*z* 271, 243, and 151. In the same manner, sinodiflavonoid B (47) was identified at *m*/*z* 603.0778 [M−H]^−^. These results are in agreement with data on the flavonol composition of *Allium cepa* [6,22,23].

Anthocyanins are characteristic of red onion varieties, giving them their unique red/purple color [24]. The most frequently reported anthocyanins in RO peels are acetylated cyanidin derivatives [22,25]. In RO peel extract, two cyanidin derivatives were tentatively identified: cyanidin-*O*-malonylhexoside (54) showed a deprotonated molecule [M−H]^−^ at *m*/*z* 533.0903 and an ion fragment detected at *m*/*z* 285 [M−H−malonylhexosyl]^−^. Moreover, the parent ion observed at *m*/*z* 489.1038 has been suggested to be cyanidin-*O*-hexoside acetate (57), whereas the ion fragment detected at *m*/*z* 284 may have been produced by the loss of the hexosyl acetate moiety [M−H−hexosyl acetate]^−^ [25]. As reported, petunidin-*O*-hexoside (58) was detected by its parent ion at *m*/*z* 479.1176, and an ion fragment was detected at *m*/*z* 317 [M−H−hexosyl]^−^; moreover, an ion fragment was detected at *m*/*z* 284 [M−H−hexosyl−H_2_O−CH_3_]^−^. In the same manner, peonidin-*O*-hexoside and delphinidin-*O*-hexoside acetate (55 and 56, respectively) were detected in both extracts [22].

Phenolic acid derivatives: The proposed method was useful for the characterization of hydroxybenzoic acid derivatives (gallic and protocatechuic acid derivatives). Protocatechuic acid (9) and its derivatives (8, 18, and 19) were detected in both RO and YO peel extracts. Gallic acid (11) and its derivatives (21 and 22) were detected mainly in RO peel extract. The parent ion observed at *m*/*z* 329.088 has been suggested to be protocatechuic acid, methyl ether, *O*-hexoside (18), where the ion fragment detected at *m*/*z* 167 may have been produced by the loss of the hexosyl moiety [M−H−hexosyl]^−^. Moreover, protocatechuic acid (9) showed a deprotonated molecule [M−H]^−^ at *m*/*z* 153.0194 and an ion fragment detected at *m*/*z* 109 [M−H−CO_2_]^−^, which have already been identified in onion peel extracts [6]. Concerning hydroxycinnamic acid derivatives, the data obtained from the Q-TOF-MS analysis showed a deprotonated molecule [M−H]^−^ at *m*/*z* 353.0873, and the neutral loss of 162 amu of the caffeoyl moiety was observed in MS^2^ fragmentation at *m*/*z* 191 in YO peel extract, which was proposed as chlorogenic acid (14). 

### 2.2. Antimicrobial Activity of Onion Peel Extracts (Minimum Inhibitory Concentration (MIC) and Minimum Bactericidal Concentration (MBC))

Onion peel extracts were found to be effective in retarding microbial growth, with a zone of inhibition ranging from 8 to 26 mm (Table 2). RO (100 mg/mL) was the most effective extract in retarding microbial growth, with zones of inhibition ranging from ~10 to 26 mm. It exhibited a significantly higher zone of inhibition than the positive control antibiotic discs (Vancomycin 30 µg, Ceftazidime 30 µg, and Diflucan 2 mg/mL). YO was effective only against *S. aureus* and MRSA, with zones of inhibition of ~15 and 16 mm, respectively. *P. aeruginosa*, and *C. albicans* were the most resistant strains to YO, while *S. epidermedis* was the least susceptible strain. The MIC values varied from 1.9 to 31.25 mg/mL. RO peel extract demonstrated the highest antibacterial activity against *S. aureus* and MRSA with the lowest MIC of 1.9 mg/mL, followed by *S. epidermidis* and *C. albicans* with an MIC of 7.8 mg/mL, while it showed the least antibacterial effect against *P. aeruginosa* with an MIC of 31.25 mg/mL. The highest antibacterial effect of YO was recorded against *S. aureus* and *C. albicans*, with an MIC of 15.6 mg/mL, followed by MRSA (MIC = 31.25 mg/mL) (Table 2). Both peel extracts demonstrated a bactericidal effect, as the MBC/MIC ratio was ≤4 [26,27].

### 2.3. Inhibition of Biofilm Formation and Destruction of Biofilm Masses 

**Figure 3:**
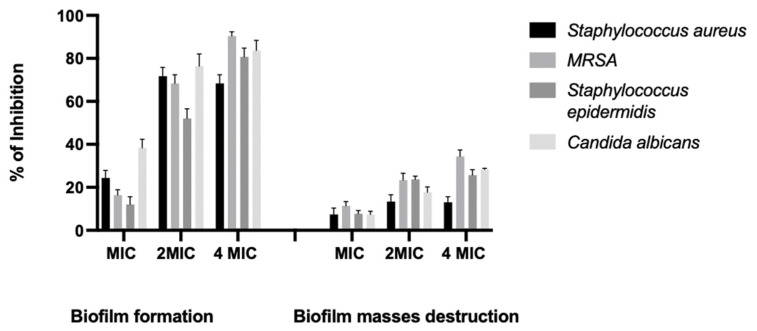
Demonstration of biofilm reduction and destructive effects of RO peel extract on microbial isolates at concentrations of MIC, 2MIC, and 4MIC.

The creation of biofilms, which is commonly observed in chronic wounds, is a fundamental factor contributing to the persistence of infections and the development of resistance to antimicrobial agents [28]. In our study, treatment of the biofilm with RO peel extract at 2MIC and 4MIC for 24 h showed good prevention of biofilm attachment, with a percentage of inhibition ranging from 70% to 91.6% against *S. aureus*, MRSA, and *C. albicans* (Table 3, Figure 3). According to established criteria [29], a natural extract resulting in inhibition above 50% is considered to have good antibiofilm activity. The ability of RO to inhibit biofilm formation makes it a promising source of drug leads to control microbial biofilm growth. RO peel extract showed a small percentage reduction of biofilm masses (12.5% to 33.33% at 2MIC and 4MIC, respectively), indicating that the inhibitory effects of the extracts were directly correlated with RO. Consequentially, RO peel extract inhibits initial biofilm formation more than disrupting mature biofilms, in accordance with a previous study [30].

### 2.4. RO Peel Extract Limits C. albicans Skin Infection and Improves Skin Appearance

**Figure 4:**
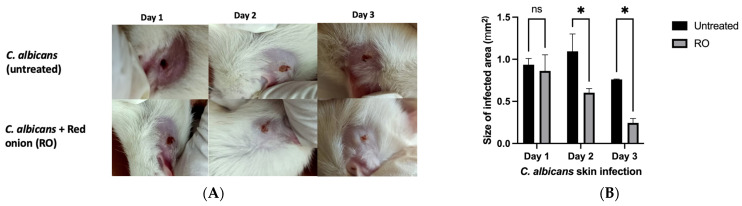
(**A**): In vivo imaging of rat skin infected with *Candida albicans*. (**B**): The size of infected skin by *C. albicans* when analyzed by ImageJ software. Significance is considered at *p* < 0.05. The quantity of asterisks (*) attached to the columns denotes the degree of significance; ns: no significance. Data are presented as mean ± SEM.

The excessive proliferation of *C. albicans* can lead to both superficial and potentially fatal systemic infections when entering the bloodstream due to the activity of candidalysin, a secreted toxin that triggers inflammatory signaling [31]. The findings of our study indicate that the application of RO peel extract on infected skin effectively restricted the development of skin infection caused by *C. albicans* when analyzed by ImageJ software as compared to the untreated group. Furthermore, this treatment demonstrated a notable improvement in the visual appearance of the skin within a three-day period (Figure 4). Additionally, the single plate–serial dilution spotting (SP-SDS) method showed that the regions of three serial dilutions (10^−1^, 10^−2^, and 10^−3^) exhibited spot growth and produced colonies within the acceptable range. The colony count technique determined the number of viable cells formed on SDA. After 72 h of incubation at 25 °C, colonies were enumerated in yeast suspensions treated with RO peel extract. In addition, the untreated (induction) rat exhibited colony growth as well as the negative control rats. The Petri dishes were stored in the incubator. After a single night of expansion, colonies were visible to the unaided eye. In the case of 10^−2^ dilutions, an average of nine colonies per location could be observed, whereas in the case of 10^−3^ dilutions, only two colonies were observed on average. In rats treated with RO peel extract, colonies were less numerous to count (LNTC) in all dilutions compared to the induction group, where colonies were too numerous to count (TNTC) and 3.2 × 10^6^ CFU/mL in 10^−1^ and 10^−3^, respectively. While in the control group, colonies were TNTC and 7.4 × 10^6^ CFU/mL in 10^−2^ and 10^−3^, respectively.

### 2.5. In Vitro Screening of the Cytotoxicity and Anti-Inflammatory Effects of Onion Peel Extracts

Human adenocarcinoma cells from the MCF-7 and HT-29 cell lines were treated with RO and YO peel extracts to investigate how they affected cell growth and survival. The findings of this study demonstrated that the extracts, at a concentration of 10 μg/mL, did not manifest any cytotoxic effects or hinder cellular proliferation in either of the cell lines. This observation suggests that the extracts possess a high level of safety. These results align with the previous research conducted by Salem et al., which also reported the greatest cell viability following treatment with onion peel extract [20]. However, at a concentration of 100 μg/mL, the extracts decreased cell viability to 90.4% and 91.6% in HT-29 cells and to 74.6% and 79.3% in MCF-7 cells for RO and YO peel extracts, respectively. In terms of anti-inflammatory activity, both RO and YO peel extracts demonstrated promising inhibition of lipopolysaccharide (LPS)-induced nitric oxide (NO) release in RAW macrophages, with maximal inhibition observed at 100 μg/mL (Table 4). According to a recent study, the anti-inflammatory activity of onion peel extract can be attributed to its elevated concentration of flavonoids, particularly quercetin, in comparison to the edible portions. Flavonoids have been observed to mitigate inflammatory diseases through the modulation of pro-inflammatory cytokine levels [32].

### 2.6. RO and YO Peel Extracts Improve Wound Healing and Tissue Repair

**Figure 5:**
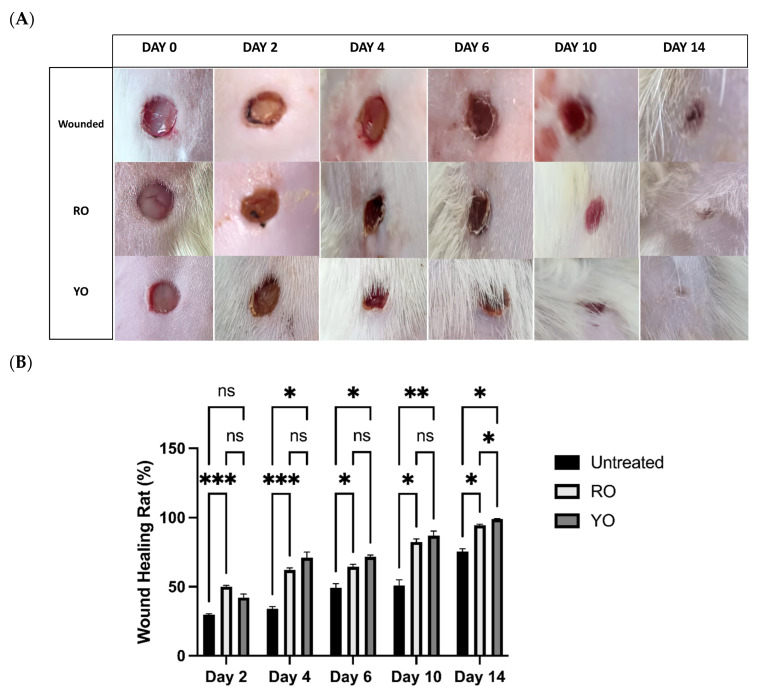
Wound healing and tissue repair in rats treated with RO and YO peel extracts. (**A**): Optical images; (**B**): Wound healing rate % at 0, 2, 4, 6, 10, and 14 days. Data are presented as mean ± SEM. Significance is considered at *p* < 0.05. The quantity of asterisks (*) denotes the degree of significance; (*): *p* < 0.05, (**): *p* < 0.01, (***): *p* < 0.001; ns: no significance.

To explore the tissue-repairing power of onion peel extracts, the gel preparation of both extracts was applied once daily as a uniform layer covering the wound area for 14 days. Figure 5 illustrates wound closure appraisal; the captured optical images were analyzed by ImageJ software. Obviously, the wound size was significantly reduced in both the RO- and YO-treated groups as compared to the untreated group. However, on day 14, the YO-treated group showed the highest repair potential as compared to the RO-treated group, since a barely visible residual scar was observed.

### 2.7. Onion Peel Extract Promotes Wound Healing and Ameliorates Histopathological Alterations in Wounded Tissues

**Figure 6:**
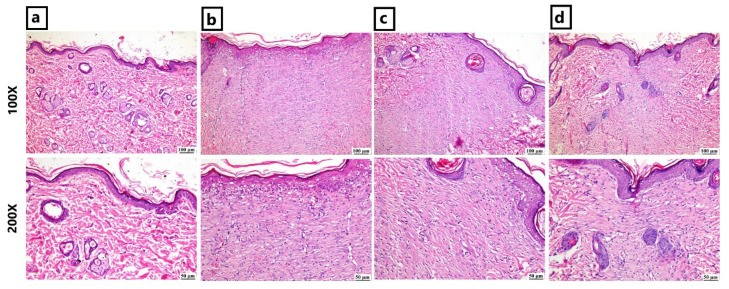
Photomicrographs of skin (H&E) showing (**a**) negative control group: normal skin; (**b**) wounded group: wound area filled with granulation tissue and complete re-epithelization; (**c**) RO-treated group: filling of wound area with collagen-rich tissue and complete re-epithelization; (**d**) YO-treated group: contracture of the wound area with complete re-epithelization.

The negative control group showed a normal histological skin structure. However, the wounded group revealed the presence of a wide area of wound filled with granulation tissue, few blood spaces, and minute areas of hemorrhage. Numerous inflammatory cells were infiltrating the wound, which was covered by a complete layer of newly formed epithelium. The RO-treated group showed better healing signs; the wound surface showed complete re-epithelization, and the inflammation subsided. The wound gap was filled with collagen-rich fibrovascular tissue. Furthermore, marked improvement was noticed in the YO-treated group due to marked contraction, resulting in a reduction in wound size and the existence of more collagen bundles at the wound area with minimal inflammatory cell infiltration. The wound surface showed complete epithelial coverage with evidence of keratinization (Figure 6).

**Figure 7:**
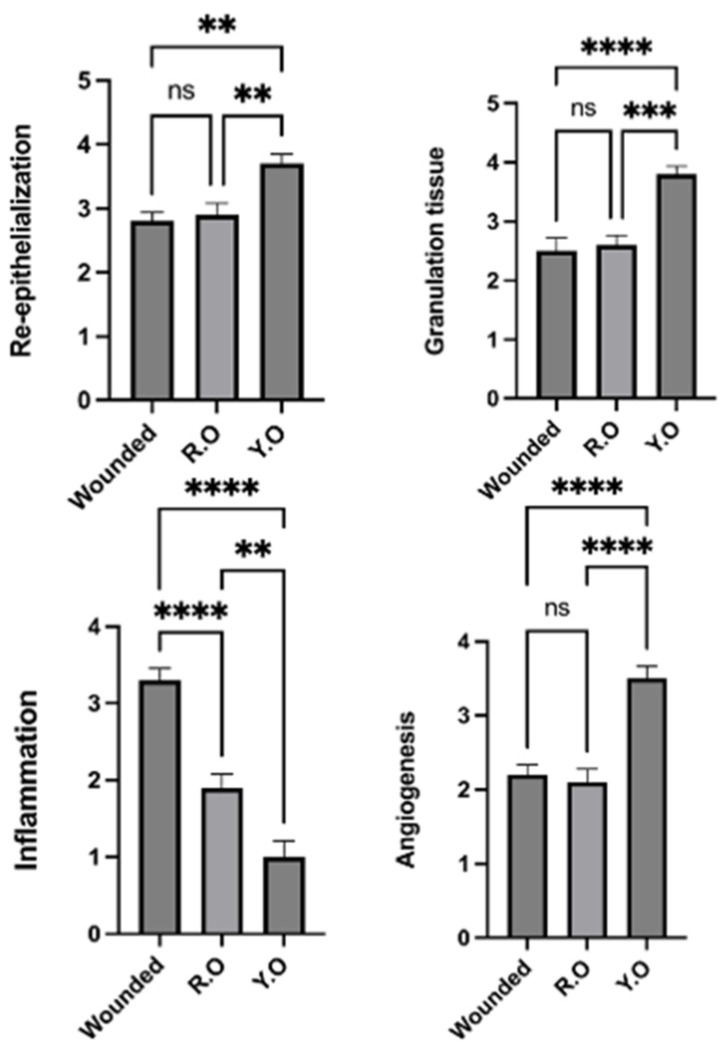
Wound healing histological score: charts represent healing criteria in each group. The negative control group showed normal skin without wound area. Data are presented as mean ± SEM. Significance is considered at *p* < 0.05. The quantity of asterisks (*) denotes the degree of significance; (**): *p* < 0.01, (***): *p* < 0.001, (****): *p* < 0.0001; ns: no significance.

As illustrated in Figure 7, significantly better re-epithelization was noticed in the YO-treated group when compared to the RO-treated group. In comparison to other groups, the YO-treated group exhibited significantly improved collagen. Both treatments significantly reduced the degree of inflammation in the wound area. Angiogenesis was significantly higher in the YO-treated group.

### 2.8. YO Peel Extract Suppresses Wound-Associated Inflammation

**Figure 8:**
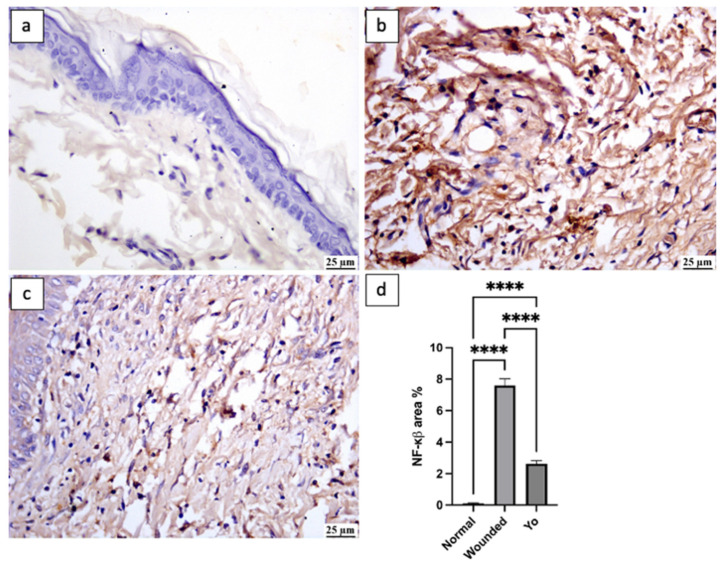
Photomicrograph of skin, immune expression of NF-κβ (immune staining). (**a**) Normal group showing limited NF-κβ expression; (**b**) wounded group showing intense NF-κβ expression; (**c**) YO-treated group showing moderate NF-κβ expression; (**d**) chart representing NF-κβ expression. Data are presented as mean ± SEM. Significance is considered at *p* < 0.05. The quantity of asterisks (*) denotes the degree of significance; (****): *p* < 0.0001; ns: no significance.

**Figure 9:**
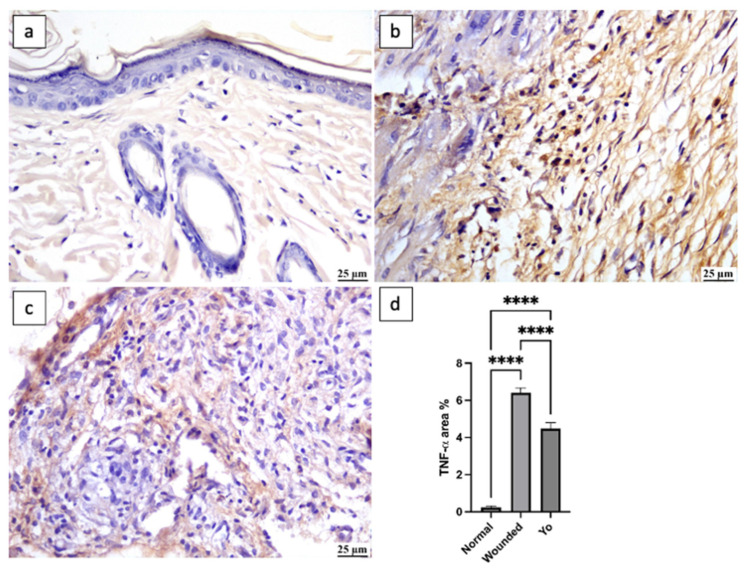
Photomicrograph of skin, immune expression of TNF-α (immune staining). (**a**) Normal group showing weak TNF-α expression; (**b**) wounded group showing increased TNF-α expression; (**c**) YO-treated group showing moderate TNF-α expression; (**d**) chart representing TNF-α expression. Data are presented as mean ± SEM. Significance is considered at *p* < 0.05. The quantity of asterisks (*) denotes the degree of significance; (****): *p* < 0.0001; ns: no significance.

**Figure 10:**
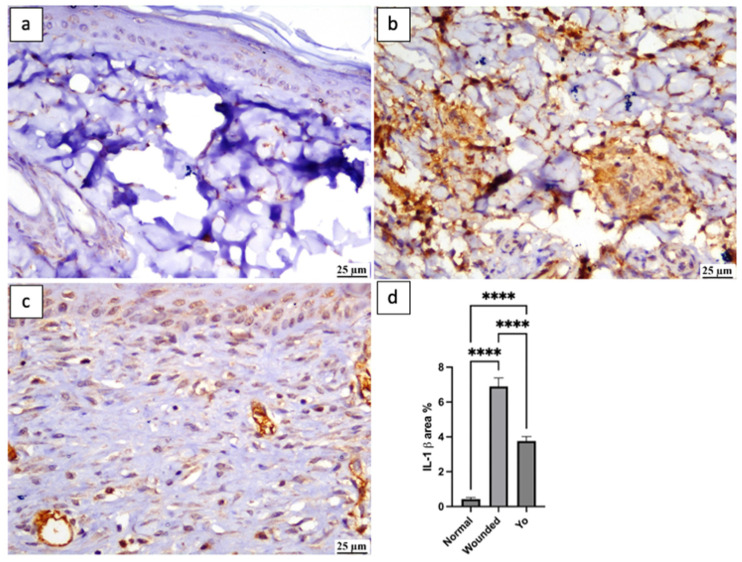
Photomicrograph of skin, immune expression of IL-1β (immune staining). (**a**) Normal group showing weak IL-1β expression; (**b**) wounded group showing intense IL-1β expression; (**c**) YO-treated group showing moderate IL-1β expression; (**d**) chart representing IL-1β expression. Data are presented as mean ± SEM. Significance is considered at *p* < 0.05. The quantity of asterisks (*) denotes the degree of significance; (****): *p* < 0.0001; ns: no significance.

Inflammation is a physiological process that emerges in response to wound injury and plays a beneficial role by aiding in the defense against invading pathogens and eliminating dead tissues from the injury site [33]. Although inflammation has a positive impact, if it persists for a prolonged time, it may slow down the healing process, resulting in extravagant scarring [34]. Therefore, it is crucial to maintain a proper balance in the inflammatory cascade to achieve relevant healing. In this study, applying YO peel extract significantly suppressed inflammatory markers NF-κβ, TNF-α, and interleukin-1 beta (IL-1β) by 65%, 30%, and 54%, respectively, as compared to the untreated group (*p* < 0.05) (Figure 8, Figure 9 and Figure 10), which is consistent with the previous study of Kim et al. [35].

### 2.9. YO Peel Extract Accelerates Angiogenesis and Curtails Notch-1 Expression

**Figure 11:**
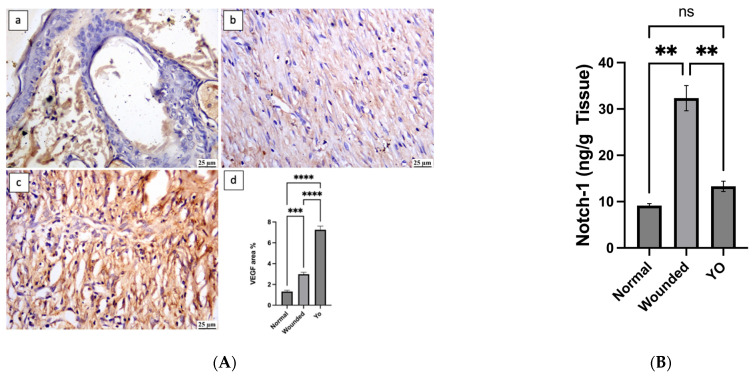
(**A**): Photomicrograph of skin, immune expression of VEGF (immune staining). (**a**) Normal group showing mild VEGF expression; (**b**) wounded group showing moderate VEGF expression; (**c**) YO-treated group showing intense VEGF expression; (**d**) chart representing VEGF expression. (**B**): Notch-1 levels (ng/g tissue) in normal, wounded, and YO-treated groups. Data are presented as mean ± SEM. Significance is considered at *p* < 0.05. The quantity of asterisks (*) denotes the degree of significance; (**): *p* < 0.01, (***): *p* < 0.001, (****): *p* < 0.0001; ns: no significance.

Vascular endothelial growth factor (VEGF) is one of the positive regulators in the angiogenesis process, which plays a crucial role in wound healing [36]. In comparison with the wounded group, the YO-treated group showed accelerated angiogenesis, as reflected in the significant elevation in VEGF expression at the wound area (Figure 11). This explains the efficacy of YO peel extract in repairing wounded tissue owing to the flavonoids and fatty acid content, which can facilitate the growth of new tissue by providing wounds with essential nutrients and newly generated cells [37]. Additionally, according to reports, the activation of Notch-1 signaling can slow the healing of wounds by inhibiting angiogenesis and triggering the inflammatory cascade [16,38]. This is consistent with our findings since the Notch-1 levels in skin tissue homogenate were significantly higher in the wounded group than in the normal group (*p* < 0.05). On the other hand, YO peel extract application to wounds dramatically decreased Notch-1 levels in comparison to the wounded group (*p* < 0.05).

### 2.10. YO Peel Extract Inhibits NLRP3 Inflammasome and Caspase-1 Signaling

**Figure 12:**
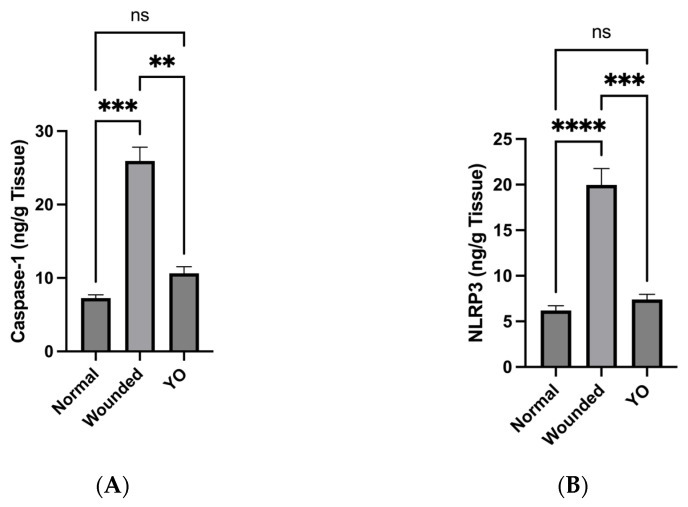
(**A**): Caspase-1 level (ng/g tissue) in normal, wounded, and YO-treated groups. (**B**): NLRP3 level (ng/g tissue) in normal, wounded, and YO-treated groups. Data are presented as mean ± SEM. Significance is considered at *p* < 0.05. The quantity of asterisks (*) denotes the degree of significance; (**): *p* < 0.01, (***): *p* < 0.001, (****): *p* < 0.0001; ns: no significance.

Increased inflammatory responses are caused by the long-term activation of the Nod-like receptor protein (NLRP) inflammasome within wounds, which can worsen damage to the wound [39]. Thus, targeting NLRP3 downstream signaling can improve wound healing [40]. Our results revealed the efficacy of YO peel extract in downregulating the levels of caspase-1, one of the inflammatory-mediated caspases, and NLRP3 significantly as compared to the wounded group (Figure 12).

Hence, the differential activity found among onion types may be attributable to their asymmetrical chemical compositions. Notably, RO peel extract is characterized by a substantial presence of anthocyanins. RO peel extract completely stopped biofilm from forming and stopped *C. albicans* from infecting the skin. This can be attributed to the high concentration of flavonoids and phenolics. Consequently, RO peel extract showed great potential as a valuable resource for the development of novel drugs aimed at controlling microbial growth. On the other hand, YO peel extract has been found to have beneficial effects on tissue regeneration and wound healing. It achieves this by reducing inflammation mediated by NLRP3/caspase 1 signaling while also increasing angiogenesis and suppressing levels of Notch-1. These combined effects contribute to improved tissue regeneration and wound healing, with the added benefit of reduced scar formation.

## 3. Materials and Methods

### 3.1. Chemicals

Dimethyl sulfoxide (DMSO) was provided by Sigma Aldrich (St. Louis, MO, USA). Hydroxypropyl methyl cellulose (HPMC) K4M was from Colorcon (Kent, UK). Other chemicals were of the highest purity.

### 3.2. Plant Material and Extraction

Onions (*Allium cepa* L.) of the red and yellow varieties were purchased at the ripe stage from a local market in Egypt (June 2021). Their identity was confirmed in the pharmacognosy department, Faculty of Pharmacy, O6U, Giza, Egypt. The outer dry protective layers of the onion bulbs were carefully separated, powdered (500 g each), and extracted separately in Soxhlet with methanol (80%, 2 × 500 mL, 65 °C, 1 h). Filtered extracts were evaporated individually under vacuum (Rotavapor^®^, BÜCHI, Flawil, Switzerland) [21] and used for biological and chemical investigations.

### 3.3. UPLC-QTOF–MS/MS Analysis

Ultra-high-performance liquid chromatograms (UPLCs) were obtained on an Agilent LC-MS system composed of an Agilent 1290 Infinity II UPLC coupled to an Agilent 6545 ESI-Q-TOF-MS in negative ionization mode. Aliquots (1 µL) of RO and YO peel extracts (1 mg/mL in MeOH) were analyzed on a Kinetex phenyl-hexyl (1.7 µm, 2.1 × 50 mm) column eluted with 1 min isocratic elution of 90% A (A: 100% H_2_O + 0.1% formic acid) followed by 6 min linear gradient elution to 100% B (95% MeCN + 5% H_2_O + 0.1% formic acid) with a flow rate of 0.4 mL/min. ESI conditions were set with the capillary temperature at 320 °C, source voltage at 3.5 kV, and a sheath gas flow rate of 11 L/ min. Ions were detected in the full scan at an intensity above 1000 counts at 6 scans/s, with an isolation width of 1.3 ~*m*/*z*, a maximum of 9 selected precursors per cycle, and using ramped collision energy (5 × *m*/*z*/100 + 10 eV) [41]. The acquired MS/MS data were processed as Agilent Mass Hunter data files (.d).

### 3.4. Antimicrobial Activity Evaluation

#### 3.4.1. Microbial Strain and Inoculum Preparation

Skin pathogenic microbial strains, *Staphylococcus aureus* (ATCC 25923), Methicillin-Resistant *Staphylococcus aureus* (MRSA, ATCC 20213), *Staphylococcus epidermidis* (ATCC 12228), *Pseudomonas aeruginosa* (ATCC 9027), and a fungal pathogen strain, *Candida albicans* (ATCC 10231), were provided from the Microbiology and Immunology Department, Faculty of Pharmacy, O6U, Giza, Egypt. Skin pathogenic microbes were subcultured aerobically overnight (37 °C) in Mueller–Hilton agar slants, except for *C. albicans*, which was planted in a Sabouraud agar slant (25 °C). The microbial growth was harvested using sterile saline water and diluted to adjust the optical density of a 0.5 McFarland standard (1.5 × 10^7−8^ CFU/mL). All antimicrobial experiments were performed in triplicate [42].

#### 3.4.2. Sensitivity Test

The antibacterial activity was accessed using the disc diffusion method. RO and YO peel extracts (stock solution of 100 mg/mL in DMSO) were sterilized (Millipore filter 0.22 mm) and then loaded over sterile filter papers (4 mm diameter) on Petri dishes containing microbial strains individually. After incubation, the inhibition zones (mm) were measured. Antibiotic discs were used as a positive control, and DMSO-loaded discs were used as a negative control [7].

#### 3.4.3. MIC and MBC Determination by Microtiter Broth Dilution Method

The MIC of the RO and YO peel extracts was determined by the serial microdilution method [27] according to the Clinical and Laboratory Standards Institute guidelines. Serial dilutions (500 to 0.24 mg/mL) were performed using Mueller–Hinton broth in 96-well microplates. The bacterial suspension (5 × 10^6^ CFU/mL, 100 μL) was inoculated into each well. After incubation, the lowest concentration inhibiting bacterial or yeast growth was taken as the MIC value. From the well representing the MIC value and the two wells above it, ten microliters were taken and spread on Mueller–Hinton agar (MHA) plates. The number of colonies was counted after incubation (18–24 h, 37 °C). The concentration of the sample that produced <10 colonies was considered the MBC value [43].

#### 3.4.4. Anti-Biofilm Assay of RO Peel Extract

##### Effect of RO Peel Extract on Initial Adherence (Biofilm Formation)

Inhibition of the initial cell attachment by RO peel extract was assessed [30], and three appropriate concentrations (MIC, 2 MIC, and 4 MIC mg/mL) of the extract were prepared in Tryptic Soy Broth (TSB) medium. Of each concentration, 100 μL was transferred into wells, followed by the addition of TSB cultures (100 μL). After incubation, the plates were dried, and the biofilms were stained with crystal violet (0.1%, 15 min.). After washing with phosphate-buffered saline (PBS), the wells were filled with ethanol (96%) and incubated for 20 min, and the absorbance was measured at 595 nm using a microplate reader. The inhibition (%) of biofilm formation = [(OD negative control − OD sample)/OD negative control] × 100 [44]. Three control wells—wells containing tested bacteria + TSB (negative control), wells containing TSB + extract concentration (extract control), and wells containing TSB (media control)—were maintained for each test batch.

##### Effect of RO Peel Extract on Preformed Biofilm

Biofilms were pre-formed in microtiter plates by aliquoting 100 μL of microbes (1.0 × 10^6^ CFU/mL) into the wells and then incubating for 48 h at 37 °C. Following incubation, RO peel extract (100 μL) that showed some degree of inhibiting cell attachment was added at MIC, 2 MIC, and 4 MIC in the wells. Saline was used as the negative control. The plates were further incubated for 24 h at 37 °C. Following the incubation, crystal violet staining was performed to assess the biofilm masses.

#### 3.4.5. SP-SDS Method for Pure Yeast Cultures and Composite Samples

A total of 10 μL of *C. albicans* (0.5 McFarland) suspension was the skin of Male Dawley rats after the backs of their ears were shaved [45]. RO peel extract was applied topically 24 h later for four consecutive days to the treated group to be compared with the untreated one. The rats were sacrificed under deep anesthesia, and the infected skin was carefully isolated. The infected epidermis was immediately placed in Sabouraud broth. The solution was vortexed (15 min), and saline was added (0.9%, 1–9 mL), labelled as having a concentration of 10^−1^ milliliters. Three repeated test tubes containing saline solution produced 10^−2^ and 10^−3^ decimal serial dilutions. To conduct SP-SDS, 9 sectors were drawn on the rear of 9 cm Petri dishes containing Sabouraud dextrose agar (SDA) material. Using a calibrated micropipette, aliquots (10 µL) of three selected dilutions were applied to these delineated areas as micro-drops. The same tip was utilized during the sample location, beginning with the lowest dilution. The plates were exposed to the laminar airflow cabinet to enable the droplets to dry, sealed in polypropylene covers, and incubated at 35 °C inverted, as required for *C. albicans*. Labeling colonies counted CFUs on the plate’s reverse. The pattern of colony development at different dilutions in SP-SDS was recorded as spot growth; counts greater than 250 were considered TNTC because determination was impossible; plates with less than 25 colonies were considered LNTC and did not have a statistically significant number of colonies or acceptable colonies (25–250 range). After noting the dilution level yielding viable colonies and the CFU per sector, the CFU per 10 μL was calculated as follows: for instance, if 25 colonies grew for the 10^−6^ dilution plated with 10 μL, the CFU/mL would be 2.5 × 10^9^, [(25 × (1/10^−6^) × 1000)/10] = 2.5 × 10^9^ CFU/mL [46].

### 3.5. Pharmacological Activity Evaluation

#### 3.5.1. In Vitro Sulforhodamine B (SRB) Cytotoxicity Assay

Evaluating the effect of onion peel extracts on cell viability was assessed by the SRB assay [47]. The cell lines utilized in this study were MCF-7, derived from breast adenocarcinoma, and HT-29, derived from colorectal cancer. These cell lines were obtained from the American Type Culture Collection (ATCC) and cultured in DMEM and RPMI medium, respectively. The assessment of cell viability was conducted at two different doses, specifically 10 and 100 µg/mL of RO and YO extracts. Each extract was evaluated individually. The cell viability % = [Absorbance of treated cells/Absorbance of control cells] × 100. All assays were performed in triplicate.

#### 3.5.2. In Vitro Anti-Inflammatory Screening

Murine macrophage RAW264.7 cells (ATCC) were seeded into 96-well microwell plates and incubated overnight. The next day, RO and YO peel extracts (10 and 100 μg/mL in DMSO separately) were added to the cells after inducing inflammation by LPS at 100 ng/mL in a complete culture medium. After incubation (24 h), Griess assay was used to determine nitric oxide (NO) [48]. The absorbance was measured on a Tecan Sunrise™ microplate reader (540 nm) (Austria). NO inhibition % of RO and YO peel extracts was calculated relative to the inflammation group, normalized to cell viability determined with the Alamar Blue™ reduction assay [49].

#### 3.5.3. Evaluating Excisional Wound Healing Activity In Vivo

##### Preparation and Characterization of Onion Peel Extract Gel for Topical Application

RO and YO peel extracts were transformed into gel to augment skin retention during their application to skin wounds. Briefly, hydroxypropyl methylcellulose (HPMC) at a concentration of 2% *w*/*w* was mixed with each extract upon the formation of gel using a magnetic stirrer. The appearance, precipitation, and homogeneity of freshly fabricated gels were visually assessed using the black and white surface [50], and there was no aggregation or precipitation; also, the consistency of all the generated gels was homogeneous.

##### Animals and an Excisional Wound Model

Male Dawley rats aged 10 weeks (300–320 g) obtained from the Nile Co. for pharmaceutical and chemical industries, Cairo, Egypt, were used in the experiments. Animals were housed (4/cage) under standard housing conditions (25 ± 1 °C, 12 h light/dark cycles). Before any experimental treatments, rats were left to cope for 1 week, allowing free access to standard diet pellet chow and tap water. The experimental protocol adhered to NIH guidelines and was approved by the O6U Research Ethics Committee (PRE-Ph-2303005). An excision wound was created for all groups except for the negative control group [51]. Briefly, dorsal hair for all groups was shaved before wound incision, the skin was disinfected (alcohol 70%), and then, after deep anesthesia, a circular wound with a diameter of 10 ± 2 mm was performed using a skin biopsy tool and surgical scissors. The animals were divided into 4 groups (8 rats each): GPI (negative control); GPII (positive control); GPIII, a treatment group receiving RO peel extract gel (60 mg/day); and GPIV, a treatment group receiving YO peel extract gel (60 mg/day). Treatment was initiated 24 h after the wound was created. Wound shrinkage and repair were measured visually and digitally using a digital camera and an image analyzer (Image J.2.0 software, NIH, New York, NY, USA) at 0, 2, 4, 6, 10, and 14 days after the wound excision. The progression of wound healing (%) was calculated: (initial wound area–current wound area)/initial wound area × 100. At the end of the experiment, animals were sacrificed by cervical dislocation after deep anesthesia using isoflurane. From three animals from each group, the dorsal skin was taken and fixed in paraformaldehyde (4%) for histopathological examination and immunohistochemistry analysis of VEGF, NF-κb, TNF-α, and IL-1β. For the rest of the animals in all groups, the skin of the wound area was removed and homogenized in PBS for evaluating the protein levels of Notch-1, caspase-1, and NLRP3 by ELISA [51].

##### Histopathological Evaluation

Fixed skin tissue samples were exposed to different concentrations of alcohol and xylene before being immersed in melted paraffin and stained with hematoxylin and eosin (H&E) [52]. Under a light microscope, the progression of wound healing was analyzed as previously outlined by Bakr et al. [53]. Briefly, the degree and quality of re-epithelialization and the degree of organization of granulation tissue production were described by numbers ranging from 0 to 4. The reduction in the number of inflammatory cells per microscopic area was assigned a number between 0 and 4 to represent the degree of inflammation. Angiogenesis was also assessed microscopically by counting the number of vessels per site. Additionally, collagen content was assessed by staining tissue sections with the Masson trichrome stain (MTC) and quantifying MTC-stained areas as (area%) using Cellsens dimensions.

##### Immunohistochemistry

Sections (5 µm) were cut for immune staining. The tissue sections were incubated with primary antibodies of IL-1 β, TNF- α, NF-κβ, and VEGF (anti-IL-1β (SC-12742), anti-TNF-α (SC-52746), anti-NF-κβ (SC-8008), and anti-VEGF (SC-57496), Santa Cruz, Biotechnology, Inc., at a dilution of 1:200) for 1 h at room temperature, followed by washing with TBS. Endogenous peroxidases were blocked using hydrogen peroxide. Afterward, an HRP-labeled detection kit (Bio SB, BSB0001, USA) was used as per the manufacturer’s instructions to develop the positive reaction. Negative controls were obtained by deleting incubation with primary antibodies. Positive immune staining was quantified as area percent using LAS-X software (Leica v4.13, Germany).

##### Enzyme-Linked Immunosorbent Assay (ELISA)

Notch-1, caspase-1, and NLRP3 concentrations in skin tissue homogenate were evaluated using ELISA kits ((Notch-1) ELISA Kit, MyBiosource, Cat.No. MBS930664; Rat caspase-1 ELISA Kit, LifeSpan Bioscience, Cat.No. LS-F6716; and Rat NLR Family, (NLRP3) ELISA Kit, MyBiosource, Cat.No. MBS2033695, respectively) in accordance with the instructions provided by the manufacturer.

### 3.6. Statistical Analysis

Data were expressed as the mean ± SEM, and statistical analysis was carried out by one-way ANOVA followed by the Tukey multiple comparisons test to calculate the significance of the difference between treatments. Values of *p* < 0.05 were considered significant. All statistical analyses were performed and graphs were sketched using GraphPad Prism (ISI, USA) software (version 9). The quantity of asterisks (*) attached to the columns denoted the degree of significance; ns: no significance.

## 4. Conclusions

A thorough investigation of the phytochemical and biological characteristics of onion peels unveiled their potential effectiveness, indicating that the use of onion peel extract may be an attractive option for the treatment of wounds. This is attributed to its ability to mitigate microbial infection, alleviate inflammation by modulating NLRP3/caspase 1 signaling, and promote tissue regeneration by enhancing angiogenesis and decreasing levels of Notch-1. It is highly recommended that this study progresses to clinical trials focusing on wound injuries, particularly those that are resistant and refractory in nature.

## Figures and Tables

**Table 1:** Tentatively identified metabolites via UPLC-QTOF–MS/MS in the RO and YO using negative ionization mode.

No.	*R_t_*	Proposed Compounds	Formula	[M−H] *m*/*z*	Diff. (ppm)	MS^2^ (Characteristic Fragments)	Plant
	Organic acids						
1	0.25	Dihydroxybutanoic acid	C_4_H_8_O_4_	119.0361	−9.03	101, 91	RO
2	0.53	Citric acid/Isocitric acid	C_6_H_8_O_7_	191.0198	−0.55	155, 129, 111, 67	YO
3	1.35	Quinic acid	C_7_H_12_O_6_	191.0558	1.89	173, 127, 93	YO
	Sugar derivatives						
4	0.4	Hexitol	C_6_H_14_O_6_	181.0719	−0.94	81, 71, 59	YO
5	0.4	Tetrahexoside	C_24_H_42_O_21_	665.2126	2.97	383, 179, 101, 89	RO
6	0.43	Hexuronic acid	C_6_H_10_O_7_	193.0355	−0.41	113, 85, 71, 59	RO, YO
7	0.47	Dihexoside	C_12_H_22_O_11_	341.1089	−0.51	101, 89, 71, 59	RO
	Phenolic acid derivatives						
8	0.6	Protocatechuic acid-*O*-hexoside	C_13_H_16_O_9_	315.0723	−0.54	225, 195, 153, 109	RO, YO
9	0.9	Protocatechuic acid	C_7_H_6_O_4_	153.0194	−0.47	109, 81, 53	RO, YO
10	1.12	Hydroxybenzoic acid	C_7_H_6_O_3_	137.0242	4.64	119, 93	YO
11	1.19	Gallic acid	C_7_H_6_O_5_	169.0134	4.99	151, 125, 81	RO
12	1.28	Catechol	C_6_H_6_O_2_	109.0297	−1.84	91, 81, 53	RO, YO
13	1.28	Methoxybenzoic acid	C_8_H_8_O_3_	151.0396	5.16	136, 108, 91	YO
14	1.31	Chlorogenic acid	C_16_H_18_O_9_	353.0873	1.02	191, 173	YO
15	1.51	Vanillic acid	C_8_H_8_O_4_	167.0347	2.03	152, 137, 108, 91	RO, YO
16	1.52	Methyl methoxybenzoate	C_9_H_10_O_3_	165.0549	4.76	150, 121	YO
17	1.57	Dimethoxybenzoic acid	C_9_H_10_O_4_	181.0504	1.62	166, 153, 122	YO
18	1.6	Protocatechuic acid, methyl ether, *O*-hexoside	C_14_H_18_O_9_	329.088	−0.75	167, 149, 137	RO, YO
19	1.97	Protocatechuic acid, *O*-[protocatechuyl-*O*-hexoside]	C_20_H_20_O_12_	451.0875	1.54	153, 109	YO
20	2.02	Methyl benzoate	C_8_H_8_O_2_	135.0444	5.49	122, 107, 92	YO
21	2.44	Galloyl hexoside	C_13_H_16_O_10_	331.0674	0.16	169, 151, 125	YO
22	2.47	Methyl gallate	C_8_H_8_O_5_	183.03	−0.24	165, 152, 139	RO
	Flavonoids						
23	1.5	Kaempferol-*O*-dihexoside	C_27_H_30_O_16_	609.1452	1.47	285, 259	RO, YO
24	1.86	Taxifolin-*O*-hexoside	C_21_H_22_O_12_	465.1048	0.18	303, 285, 177, 166, 151	RO, YO
25	1.9	Kaempferol-*O*-hexoside	C_21_H_20_O_11_	447.0937	0.07	285, 257	RO, YO
26	2.11	*O*-Galloylmyricitrin	C_28_H_24_O_16_	615.0994	−0.16	299, 287, 163	RO, YO
27	2.44	Dihydroxyquercetin	C_15_H_10_O_9_	333.0248	1.54	315, 287, 259, 163, 151	RO
28	2.51	Quercetin-*O*-dihexoside	C_27_H_30_O_17_	625.1421	−1.39	463, 301, 179, 151	RO, YO
29	2.63	Isorhamnetin-*O*-dihexoside	C_28_H_32_O_17_	639.1568	0.14	477, 315, 285, 151	RO, YO
30	2.71	Quercetin-*O*-hexoside-*O*-acetylhexoside	C_29_H_32_O_18_	667.15	0.85	463, 301, 245	RO
31	2.74	Quercetin, *O*-(*O*-malonyl-hexoside), *O*-hexoside	C_30_H_32_O_20_	711.1403	−0.73	463, 301	RO
32	2.74	Myricetin methyl ether	C_16_H_12_O_8_	331.0463	−0.76	207, 151, 123	YO
33	2.83	Isorhamnetin-*O*-galloylhexoside	C_29_H_26_O_16_	629.1145	1.18	467, 313, 285, 163	RO, YO
34	2.89	Taxifolin/Dihydroquercetin	C_15_H_12_O_7_	303.0515	−0.85	285, 177, 151, 125	RO, YO
35	2.94	Protocatecoyl quercetin dimer	C_22_H_14_O_11_	453.0469	−3.39	409, 301, 283, 229, 163	RO, YO
36	3.0	Taxifolin methyl ether	C_16_H_14_O_8_	333.0617	0.07	285, 243, 165, 151, 137	RO
37	3.07	Quercetin-*O*-hexoside	C_21_H_20_O_12_	463.0896	−2.49	301, 179, 151	RO, YO
38	3.21	Myricetin	C_15_H_10_O_8_	317.0292	3.34	271, 163, 152, 125	RO, YO
39	3.22	Isorhamnetin-*O*-hexoside	C_22_H_22_O_12_	477.1035	0.97	314, 299, 271, 179, 151	RO
40	3.23	Quercetin	C_15_H_10_O_7_	301.0350	0.19	273, 245, 179, 151, 121, 107	RO, YO
41	3.32	Quercetin-*O*-acetylhexoside	C_23_H_22_O_13_	505.0986	0.3	343, 301, 179, 151, 121	RO
42	3.32	Tetrahydroxyflavan, methyl ether, *O*-hexoside	C_22_H_26_O_10_	449.1448	1.22	287, 243, 228	RO
43	3.33	Trihydroxyflavan-*O*-hexoside	C_21_H_24_O_9_	419.1349	−0.37	257, 213, 195	RO
44	3.33	Quercetin-*O*-hexoside (isomer 2)	C_21_H_20_O_12_	463.0896	−2.49	301, 179, 151	RO
45	3.49	*O*-Galloylquercitrin	C_21_H_20_O_12_	463.0883	0.34	447, 301, 273, 179, 151	RO
46	3.54	Morin	C_15_H_10_O_7_	301.0345	3.01	273, 245, 179, 151, 121, 107	RO, YO
47	3.61	Sinodiflavonoid B	C_30_H_20_O_14_	603.0778	0.32	301, 273, 179, 151	RO, YO
48	3.91	Kaempferol	C_15_H_10_O_6_	285.0402	1.07	257, 229, 211, 151	RO, YO
49	3.94	Isorhamnetin	C_16_H_12_O_7_	315.0508	1.22	300, 271, 151, 107	RO, YO
50	4.06	Trihydroxyflavan	C_15_H_14_O_4_	257.0819	0.14	229, 213, 195, 151, 107	RO, YO
51	4.32	*Allium* quercetin dimer (isomer 1)	C_30_H_18_O_14_	601.0625	1.08	299, 271, 243, 151	RO, YO
52	4.58	*Allium* quercetin dimer (isomer 2)	C_30_H_18_O_14_	601.0625	1.39	299, 271, 243, 151	RO, YO
53	4.74	*Allium* quercetin dimer (isomer 3)	C_30_H_18_O_14_	601.0625	4.94	299, 271, 179, 121	RO, YO
	Anthocyanins						
54	1.22	Cyanidin-*O*-malonylhexoside	C_24_H_22_O_14_	533.0903	5.69	465, 285, 241, 107	RO
55	1.4	Peonidin-*O*-hexoside	C_20_H_30_O_12_	461.1658	2.08	299, 269, 251	RO, YO
56	2.42	Delphinidin-*O*-hexoside acetate	C_23_H_24_O_13_	507.1137	1.45	489, 285, 241	RO
57	2.65	Cyanidin-*O*-hexoside acetate	C_23_H_22_O_12_	489.1038	0.21	284, 257, 241	RO
58	2.74	Petunidin-*O*-hexoside	C_22_H_24_O_12_	479.1176	4.38	317, 299, 284	RO
	Fatty acids						
59	3.77	Trihydroxy-Octadecenoic acid	C_18_H_34_O_5_	329.2331	0.34	293, 257, 229, 211, 171	RO, YO
60	4.82	Dihydroxy-Octadecanoic acid	C_18_H_36_O_4_	315.2525	5.23	297, 267, 235, 171	YO
61	5.48	Hydroxy-Pentadecanoic acid	C_15_H_30_O_3_	257.213	−2.49	259, 217, 179	YO
62	5.56	Dihydroxy-Octadecatrienoic acid	C_18_H_30_O_4_	309.2078	−1.9	269, 241, 169, 155	YO
63	5.73	Hydroxy-Palmitic acid	C_16_H_32_O_3_	271.2275	1.48	253, 225	YO
64	6.11	Linoleic acid	C_18_H_32_O_2_	279.2324	2.47	259, 239, 219	RO, YO
65	6.24	Palmitic acid	C_16_H_32_O_2_	255.2327	0.84	235, 215, 171	RO, YO
66	6.34	Oleic acid	C_18_H_34_O_2_	281.2494	−3.36	261, 241, 129, 66	RO, YO
67	7.86	Behenic acid	C_22_H_44_O_2_	339.3263	1.62	319, 279	RO, YO

**Table 2:** Zone of inhibition, MIC, MBC, and MBC/MIC ratio of RO and YO peel extracts against the microbial isolates.

Microbial Isolates	RO Peel Extract (mg/mL)				YO Peel Extract (mg/mL)			
	Inhibition (mm)	MIC	MBC	MBC/MIC Ratio	Inhibition (mm)	MIC	MBC	MBC/MIC Ratio
*P. auruginosa*	12	31.25	62.5	2	--	-	-	-
*S. aureus*	20	1.9	1.9	1	15	15.6	15.6	1
*S. epidermidis*	13	7.8	31.25	4	8	-	-	-
*MRSA*	26	1.9	1.9	1	16	31.25	31.25	1
*C. albicans*	12	7.8	15.6	2	9	15.6	15.6	1

There was no zone of inhibition noticed with DMSO.

**Table 3:** Percentages of the inhibitory effect of RO peel extract on biofilm formation and mature biofilm masses.

Microbial Strains	Inhibition % of Biofilm Formation			Destruction % of Biofilm Masses		
	MIC	2MIC	4MIC	MIC	2MIC	4MIC
*S. aureus*	25%	70%	85%	6%	12.5%	15%
*MRSA*	16%	66.7%	91.6%	10%	25%	33.3%
*S. epidermidis*	10%	50%	80%	4%	20%	24%
*C. albicans*	37.5%	75%	85%	5%	17.5%	27.5

**Table 4:** The effects of RO and YO peel extracts on cell proliferation and NO release using in vitro screening.

Extract	Concentration (μg/mL)	Cell Viability (%)		NO Inhibition (%)
		MCF-7	HT-29	
RO	10	79.89 ± 1.6	102.13 ± 0.5	27.4 ± 0.6
	100	74.57 ± 1.5	90.35 ± 1.5	75.3 ± 3
YO	10	100.45 ± 1.3	102.03 ±0.8	25.7 ± 1.5
	100	79.3 ± 1.7	91.59 ± 1.0	62.7 ± 1.2

Data are presented as mean ± SEM.

## Data Availability

Data are contained within the article.
